# Chemokine Receptor-Specific Antibodies in Cancer Immunotherapy: Achievements and Challenges

**DOI:** 10.3389/fimmu.2015.00012

**Published:** 2015-01-30

**Authors:** Maria Vela, Mariana Aris, Mercedes Llorente, Jose A. Garcia-Sanz, Leonor Kremer

**Affiliations:** ^1^Department of Immunology and Oncology, Centro Nacional de Biotecnología, Consejo Superior de Investigaciones Científicas (CNB/CSIC), Madrid, Spain; ^2^Centro de Investigaciones Oncológicas, Fundación Cáncer, Buenos Aires, Argentina; ^3^Protein Tools Unit, Centro Nacional de Biotecnología, Consejo Superior de Investigaciones Científicas (CNB/CSIC), Madrid, Spain; ^4^Department of Cellular and Molecular Medicine, Centro de Investigaciones Biológicas, Consejo Superior de Investigaciones Científicas (CIB/CSIC), Madrid, Spain

**Keywords:** chemokine receptors, therapeutic antibodies, clinical trials, immunotherapy, cancer

## Abstract

The 1990s brought a burst of information regarding the structure, expression pattern, and role in leukocyte migration and adhesion of chemokines and their receptors. At that time, the FDA approved the first therapeutic antibodies for cancer treatment. A few years later, it was reported that the chemokine receptors CXCR4 and CCR7 were involved on directing metastases to liver, lung, bone marrow, or lymph nodes, and the over-expression of CCR4, CCR6, and CCR9 by certain tumors. The possibility of inhibiting the interaction of chemokine receptors present on the surface of tumor cells with their ligands emerged as a new therapeutic approach. Therefore, many research groups and companies began to develop small molecule antagonists and specific antibodies, aiming to neutralize signaling from these receptors. Despite great expectations, so far, only one anti-chemokine receptor antibody has been approved for its clinical use, mogamulizumab, an anti-CCR4 antibody, granted in Japan to treat refractory adult T-cell leukemia and lymphoma. Here, we review the main achievements obtained with anti-chemokine receptor antibodies for cancer immunotherapy, including discovery and clinical studies, proposed mechanisms of action, and therapeutic applications.

## Introduction

Cancer treatment is expanding from non-specific cytotoxic chemotherapies to targeted therapies. These are focused on fighting cancer cells, modifying the tumor microenvironment, or enhancing anti-tumor immunity (1–3). Malignant and stromal cells secrete a variety of proteins, including matrix components, proteolytic enzymes, growth factors, pro-inflammatory cytokines, and chemokines (2, 4). Among them, tumor-associated chemokines play a central role in cancer biology, favoring leukocyte infiltration, promoting tumor growth, angiogenesis, and immune evasion (5–10).

Many tumor cells over-express functional chemokine receptors, undetectable on their normal counterparts. These receptors respond to chemokine signals by promoting cell survival, proliferation, adhesion, or migration, but also direct metastasis formation on tissues or organs where the corresponding ligands are secreted (9). Since the most common cause of death in cancer patients are metastases (11, 12), chemokine receptors expressed on the surface of cancer cells are considered suitable targets for the generation of new anti-tumor drugs (8, 13–17). As a consequence, a great effort has been made in the investigation of new drugs targeting chemokine receptors for cancer treatment. The initial focus was on the development of small molecules able to inhibit chemokine receptor signaling, although with limited success (18, 19). Recent achievements in the use of monoclonal antibodies (mAbs) targeting a variety of molecules for the treatment of leukemia, breast cancer, colon cancer, and melanoma (20–23), have contributed to move the efforts onto targeting chemokines and their receptors toward generating specific antibodies for therapeutic purposes.

The clinical use of an anti-chemokine receptor mAb, mogamulizumab, specific for the C–C chemokine receptor type 4 (CCR4) has been granted in Japan for the immunotherapy of patients with relapsed or refractory CCR4^+^ adult T-cell leukemia (ATL) (24). This antibody is currently in phase II and III clinical trials in Europe and the USA for the treatment of patients with ATL, cutaneous T-cell lymphoma (CTCL), or peripheral T-cell lymphoma (PTCL) (24, 25). In addition, antibodies specific for the CCR2 and CXCR4 receptors are also being evaluated in clinical trials, while antibodies against many other chemokine receptors have shown effectiveness in different xenograft models of cancer (26–38).

## Chemokines and Their Receptors in Cancer

Chemokines are a family of small chemotactic cytokines, with 44 members in humans, which generate soluble or immobilized gradients that direct the movement of cells (39, 40). Chemokines, by controlling leukocyte trafficking and recruitment, play a central role in homeostasis and the maintenance of innate and acquired immunity (41). They are essential in mammalian development and organogenesis, and like other cytokines stimulate cell growth, differentiation, and activation (16). Chemokines are subdivided in four major groups, namely CX3C, CXC, CC, and C, based on the number and spacing of conserved cysteine residues on their N-terminus (39, 42). These proteins are functionally known as “inflammatory” or “homeostatic,” based on whether they are released upon inflammatory stimuli or constitutively secreted by cells located in lymphoid organs, respectively (43).

The biological effects of chemokines are exerted through their interaction(s) with specific surface receptors (chemokine receptors), structurally belonging to the seven transmembrane domain G protein-coupled receptor superfamily (GPCR). The chemokine receptor family contains 24 members in humans and can be subdivided, based on the class of chemokines they bind, into four subfamilies (CX3CR, CXCR, CCR, and XCR) all of them activating G proteins, and one subfamily (ACKR), containing 6 atypical receptors, unable to activate G proteins upon ligand binding (39, 44). It is worth to note that the chemokine/chemokine receptor system has redundancy, since some particular chemokines are able to bind to multiple receptors, and vice versa (41).

Chemokines and their receptors have been implicated in the pathogenesis of many inflammatory and infectious diseases including rheumatoid arthritis, multiple sclerosis, asthma, atherosclerosis, malaria, and AIDS (15, 16, 45, 46), but also in cancer (5, 47). Expression levels of chemokines and their receptors are often deregulated in malignant cells, due, for example, to inactivation of tumor suppressor genes, constitutive activation of oncogenes, or altered expression of transcription factors (8, 48–50).

Expression of chemokines and their receptors play a dual role in tumorigenicity. On the one hand, chemokines secreted by either the cancer-initiating cells or the normal cells surrounding them can help limiting tumor development by increasing leukocyte migration toward the site, and inducing long-term anti-tumor immunity. On the other hand, they may facilitate survival, proliferation, and metastatic potential of tumor cells (6, 10, 51–54). The initially secreted chemokines at the tumor site play a key role defining the composition of the tissue stroma and recruiting tumor infiltrating leukocytes bearing specific chemokine receptors (CXCR1, CXCR2, CCR2, CCR4, or CCR5, among others) (7). Therefore, many chemokines are pro-inflammatory for most tumors (4) and have key functions in tumor angiogenesis (55, 56). For example, CXCL12 (SDF-1), the only known ligand for CXCR4, is a potent endothelial cell chemoattractant (57, 58).

Tumor cells are able to hijack the chemokine receptor/chemokine system on their own benefit. Strikingly, they “convert” infiltrating leukocytes into immuno-tolerant allies (59–61), since they are able to (i) attract suppressor T-cells and neutrophils (62–65), (ii) hijack immature dendritic cells, avoiding their migration toward the lymph nodes and therefore antigen presentation, favoring a tolerogenic profile (66), and (iii) participate in the recruitment and induction of myeloid-derived suppressor cells (67).

The most frequently over-expressed chemokine receptor in malignant cells is CXCR4 (68). It is present in over 23 different types of human cancer, including those with the highest incidence, such as lung, brain, prostate, breast, pancreas, ovarian, colorectal, leukemia, and melanomas (63). CXCR4 expression on malignant cells correlates with cell survival, tumor growth, angiogenesis, higher metastatic potential, and resistance to therapeutic agents (35, 69–76). Its ligand, CXCL12, is secreted in large amounts by bone marrow, lymph node, liver, and lung cells.

CXCR4 and CXCL12 can be used to exemplify the complexity of the chemokine/chemokine receptor networks in cancer. Unlike other chemokine receptors that have several ligands, it was originally thought that CXCL12 was the unique ligand for CXCR4 and that it was unable to bind any other receptor. In 2005, it was reported that CXCL12 was also able to bind ACKR3 (formerly CXCR7) with 10 times higher affinity (77–80). ACKR3 has two ligands CXCL12 and CXCL11. This receptor, in addition to important roles in embryonic development and cardiovascular functions (79, 81, 82), also participates in breast and lung tumorigenesis and metastasis (78). The complexity of this network is even higher since the ACKR3 ligand CXCL11 is also shared by CXCR3, a chemokine receptor with two isoforms (A and B), over-expressed in many tumors, and able to bind other ligands (CXCL9 or CXCL10) (83). Altered CXCR3 isoform expression regulates cancer cell migration and invasion (84). Indeed, CXCL11 promotes proliferative signals through binding to CXCR3-A or ACKR3, whereas binding to CXCR3-B results in growth inhibitory functions (85). The complexity of several ligands being able to bind different receptors or different isoforms of a given receptor, having different outcomes (proliferation vs. growth inhibition), should be taken into account on any pharmacological intervention.

There is broad evidence indicating that the expression of a determined chemokine receptor by a tumor preferentially directs its metastasis to the organs in which the corresponding chemokine ligand is secreted (10, 86), some of them are detailed in Table 1. Extensive revision of the expression and actions of chemokine receptors in cancer exceeds the focus of this review and can be obtained from recent reviews in Ref. (86, 87).

**Table 1:** **Human chemokine receptors and related metastases**.

Chemokine receptor^a^	Ligands^a^	Type of cancer^b^	Metastasis sites	References
CXCR1	CXCL5, CXCL6, CXCL8	Melanoma, breast cancer, ovarian cancer, prostate cancer		(88–94)
CXCR2	CXCL1, CXCL2, CXCL3, CXCL5, CXCL6, CXCL7, CXCL8	Melanoma, breast cancer, ovarian cancer, prostate, renal cell carcinoma, pancreatic cancer, esophageal cancer	Lung	(33, 88–92, 94–100)
CXCR3	CXCL4L1, CXCL4, CXCL9, CXCL10, CXCL11	Melanoma, breast cancer, colorectal cancer, osteosarcoma, ALL, B-cell CLL	Lung, lymph nodes	(101–111)
CXCR4	CXCL12	Melanoma, breast cancer, ovarian cancer, prostate cancer, glioma, neuroblastoma, squamous cell cancer, head and neck cancer, esophageal cancer, stomach cancer, bladder cancer, pancreatic cancer, colorectal cancer, renal cancer, osteosarcoma, NSCLC, AML, FCCL, ALL, CLL, NHL, multiple myeloma	Bone marrow, lymph nodes, lung, liver, peritoneum	(35, 63, 69, 70, 73, 86, 107, 110, 112–148)
CXCR5	CXCL13	Squamous cell cancer, B-cell CLL, mantle lymphoma	Bone, lymph node, peripheral nerves	(129, 149, 150)
CCR1	CCL3, CCL4, CCL5, CCL6, CCL7, CCL8, CCL13, CCL14, CCL15, CCL16, CCL23	Colorectal cancer	Liver	(151–154)
CCR2	CCL2, CCL5, CCL7, CCL8, CCL13, CCL16	Breast cancer, prostate cancer, multiple myeloma	Bone marrow, lung	(155–158)
CCR3	CCL4, CCL5, CCL7, CCL11, CCL13, CCL15, CCL24, CCL26, CCL28	Renal carcinoma, CTCL		(159, 160)
CCR4	CCL17, CCL22	CTCL, ATL, ATLL, PTCL	Skin	(161–164)
CCR5	CCL3, CCL4, CCL5, CCL7, CCL14, CCL16	Breast cancer, colorectal cancer	Liver, lung	(165, 166)
CCR6	CCL20	Colorectal cancer	Liver	(167–169)
CCR7	CCL19, CCL21	Melanoma, breast cancer, non-small cell lung cancer, lung cancer, head and neck cancer, esophageal squamous cell carcinoma, stomach cancer, gastric carcinoma, colorectal cancer, B-cell CLL, CLL	Lymph nodes	(105, 113, 115, 129, 133, 135, 136, 138, 141, 170–179)
CCR9	CCL25	Melanoma, breast cancer, prostate cancer, T-ALL	Small intestine	(86, 180–186)
CCR10	CCL27, CCL28	Melanoma, CTCL	Skin	(136, 164, 187–191)
CX3CR1	CX3CL1	Breast cancer, prostate cancer, colorectal cancer, PDAC	Bone marrow, peripheral nerves, brain	(149, 150, 192–200)
ACKR3	CCL11, CCL12	Breast cancer, NSCLC, rhabdomyosarcoma	Lung, bone marrow, liver, brain	(201–205)
ACKR6	CCL18	Breast cancer	Lung, liver	(206)

*^a^Chemokine receptors and their corresponding chemokines, following the updated nomenclature in Bachelerie et al. (39)*.

*^b^Types of cancer that over-express certain chemokine receptors*.

*ALL, acute lymphoblastic leukemia; AML, acute myeloid leukemia; ATL, adult T-cell leukemia; ATLL, adult T-cell leukemia/lymphoma; CLL, chronic lymphocytic leukemia; CTCL, cutaneous T-cell lymphoma; FCCL, follicular center lymphoma; NHL, non-Hodgkin’s lymphoma; NSCLC, non-small cell lung cancer; PDAC, pancreatic ductal adenocarcinoma; PTCL, peripheral T-cell lymphoma; T-ALL, T-cell acute lymphoblastic leukemia*.

## Antibody-Based Drugs for Cancer Therapy

Monoclonal antibodies are relatively large molecules with clear advantages for their use as therapeutic drugs. These are related to their long half-life in blood, their ability to establish specific and high affinity interactions with other molecules or with immune system cells, together with their relatively low toxicity (207–210). Drugs based on mAbs are, however, more difficult and expensive to develop and produce, and less convenient to administer than small molecule drugs. Indeed, they are able to bridge the target antigen, or cells bearing the antigen, with the innate or acquired cellular immune response (211). These characteristics, together with the development of antibody humanization techniques, phage display systems, advanced high-throughput screening methods, and transgenic mice that produce human antibodies, led to many pharmaceutical companies to invest on therapeutic mAbs. This, together with the clinical success of therapeutic antibodies during the last decade, has led to an exponential increase on the number of mAbs for cancer treatment. For instance, in oncology, chimeric and humanized mAb that entered clinical studies had approval success rates four times greater than new chemical entities, including small molecule agents (211–213). The development of therapeutic antibodies is growing fast, and includes many best-selling drugs for the treatment of cancer (rituximab, bevacizumab, trastuzumab) or immunological diseases (adalimumab, infliximab) (214).

Therapeutic antibodies for cancer treatment can be classified, on the basis of the targets they are directed to, into: (i) surface-expressed molecules on the tumor cell; (ii) cytokines, growth factors, surface receptors, or other molecules required for tumor and/or stroma proliferation or survival; and (iii) immune cell surface molecules that regulate tumor cell recognition and elimination. Conversely, on the basis of their mechanisms of action, they would be classified as mAbs that kill tumor cells through: (i) direct effects (host independent) (i.e., inhibiting receptor-ligand binding, and/or activating intracellular signaling); (ii) indirect effects (host dependent) modulating the immune response [i.e., antibody-dependent cell-mediated cytotoxicity (ADCC), complement-dependent cytotoxicity (CDC), antibody-dependent phagocytosis (ADP), etc.]; (iii) being used as molecular carriers, specifically delivering cytotoxic agents, toxins, or radio-isotopes, to target malignant cells (209); and (iv) targeting regulatory molecules on host immune system cells. Therapeutic mAbs often exert their anti-tumoral functions simultaneously using several of these mechanisms of action (29).

During the last years, a broad effort has been centered on targeting regulatory molecules from the host immune system that act as “immune checkpoints” with mAbs. Examples are ipilimumab, a mAb directed against the receptor cytotoxic T lymphocyte-associated antigen 4 (CTLA-4), or pembrolizumab (MK-3475), a mAb against the programed cell death protein-1 (PD-1), used for the treatment of metastatic melanomas (215). These mAbs inhibit the negative regulatory signals triggered by CTLA-4 or PD-1, enhancing T-cell responses against the tumor (215–218). Other mAbs, recognizing antigens on the antigen presenting cells, such as an agonist mAb against the stimulatory protein CD40 have also been postulated to harbor therapeutic effects against tumors (218, 219).

Antibodies have been generated against chemokines and their receptors. Since chemokine receptors have seven domains embedded in the cell membrane, their solubilization for obtaining the required amounts in the native conformation and the correct orientation for their use as immunogens is extremely difficult (220). These characteristics, together with their multiple post-translational modifications, low cell surface expression levels and lack of stability of their native conformations, make it particularly challenging to generate antibodies against them. Synthetic peptides had been used as immunogens, although this approach usually generates mAbs with low affinity and poor antagonistic effects (221, 222). The development of sophisticated strategies that preserve the protein native conformation during purification, along with advances in the synthesis of peptides with pre-designed structures, genetic immunization techniques, production of chemokine receptor-containing liposomes or lipoparticles, or over-expression of receptors in viral particles, enabled the generation of antibodies with higher affinities and/or able to function as strong antagonists (220). The expected *in vivo* efficacy of mAbs anti-chemokine receptors is higher than those against chemokines, since a cell surface-restricted receptor molecule is more efficiently targeted than delocalized secreted chemokines (220, 223). In addition, chemokine receptor targeting offers the possibility not only of blocking the signaling by preventing ligand binding to its receptor but also of tagging the tumor cells with the antibody, to trigger the host immune response against them.

Anti-chemokine receptor antibodies have been evaluated for the treatment of inflammatory and infectious diseases, including anti-CCR2 for rheumatoid arthritis and atherosclerosis (224); CCR3 and CCR4 for asthma and pulmonary inflammation (225–228); CXCR4 and CCR5 for HIV infections (229, 230); and CCR7 for pulmonary fibrosis (231). However, in the following paragraphs, we will only focus on their potential as anti-cancer drugs.

## Chemokine Receptors with Antibodies in Clinical Trials for Cancer Treatment

Monoclonal antibodies against CXCR4, CCR2, and CCR4 have entered clinical trials for cancer therapy. A list of trials with these antibodies is shown in Table 2, and antibodies against each of these receptors and their potential in cancer therapy are described below.

**Table 2:** **Anti-chemokine receptors antibodies for cancer therapy in clinical trials**.

mAb name; mAb class; mAb actions	Chemokine receptor	Clinical trial identifier; start date; intervention	Phase	Condition	Country	Status; completion date	Company/sponsor
MDX-1338 (232), BMS-9365649, ulocuplumab; fully human, IgG4; neutralization, apoptosis induction	CXCR4	NCT01359657^a^; 2011; monotherapy or combined with lenalidomide, dexamethasone, or bortezomib	I	Multiple myeloma	United States	Active, not recruiting	Bristol–Myers Squibb
		NCT01120457^a^; 2010; monotherapy	I	AML, DLBCL, CLL, follicular lymphoma	United States	Active, not recruiting	Bristol–Myers Squibb
MLN1202 (224); humanized, IgG1; neutralization	CCR2	NCT01015560^a^; 2010; monotherapy	II	Bone metastases	United States	Completed 2012	Millenium-Takeda/Southwestern Oncology Group
KW-0761 (233), AMG 761, mogamulizumab; humanized, IgG1; ADCC	CCR4	UMIN000010050^b^, NCT01929486^a^; 2013; monotherapy	Ia/Ib	Advanced or recurrent CCR4^−^ cancer (effect of Treg cell depletion)	Japan	Recruiting	Kyowa Hakko Kirin Pharma, Inc.
		UMIN000013294^b^; 2013; prognostic study of patient who completed Study NCT01173887^a^	II	CCR4^+^ ATLL	Japan	Not longer recruiting	Kyowa Hakko Kirin Pharma, Inc.
		NCT01626664^a^; 2012; KW0761 or investigator’s choice^d^	II	Relapsed or refractory ATLL	United States, Belgium, Brazil, France, Peru, United Kingdom	Recruiting	Kyowa Hakko Kirin Pharma, Inc.
		NCT01728805^a^; 2012; monotherapy vs. vorinostat	III	Relapsed or refractory CTCL	United States, Australia, Denmark, France, Germany, Italy, Japan, Netherlands, Spain, Switzerland, United Kingdom	Recruiting	Kyowa Hakko Kirin Pharma, Inc.
		NCT01611142^a^, 2011-004151-39^c^; 2012; monotherapy	II	Relapsed or refractory CCR4^+^ PTCL	Denmark, France, Italy, Netherlands, Spain, Germany, United Kingdom	Active, not recruiting	Kyowa Hakko Kirin Pharma, Inc.
		NCT01173887^a^; 2010; Compare mLSG15 + KW-0761 to mLSG15	II	Untreated CCR4^+^ ATLL	Japan	Completed	Kyowa Hakko Kirin Pharma, Inc.
		NCT01226472^a^; 2010; monotherapy	II	Relapsed PTCL, CTCL, including mycosis fungoides and Sezary Syndrome	United States	Completed	Kyowa Hakko Kirin Pharma, Inc.
		NCT01192984^a^; 2010; monotherapy	II	Relapsed CCR4^+^ peripheral T/NK-cell lymphoma	Japan	Completed	Kyowa Hakko Kirin Pharma, Inc.
		NCT00920790^a^; 2009; monotherapy	II	Relapsed CCR4^+^ ATLL,	Japan	Completed	Kyowa Hakko Kirin Pharma, Inc.
		NCT00888927^a^; 2009; monotherapy	I/II	Relapsed or refractory PTCL	United States	Completed	Kyowa Hakko Kirin Pharma, Inc.
		NCT00355472^a^; 2007; monotherapy	I	Relapsed or refractory CCR4^+^ ATLL, PTCL	Japan	Completed	Kyowa Hakko Kirin Pharma, Inc.

*^a^ClinicalTrials.gov Identifier*.

*^b^Unique trial number, University Hospital Medical Information Network (UMIN-CTR)*.

*^c^EudraCT number, European Union Clinical Trials Register*.

*AML, acute myelogenous leukemia; ATL, adult T-cell leukemia; ATLL, adult T-cell leukemia/lymphoma; CLL, chronic lymphocytic leukemia; CTCL, cutaneous T-cell lymphoma; PTCL, peripheral T-cell lymphoma; DLBCL, diffuse large B-cell leukemia; mLSG15 (VCAP/AMP/VECP); VCAP (vincristine sulfate, cyclophosphamide hydrate, doxorubicin hydrochloride, prednisolone); AMP, (doxorubicin hydrochloride, ranimustine, prednisolone); VECP (vindesine sulfate, etoposide, carboplatin, prednisolone)*.

*^d^Investigator’s choice of pralatrexate, or gemcitabine plus oxaliplatin, or dexamethasone plus cisplatin and cytarabine*.

### CXCR4

As demonstrated by a plethora of publications, CXCR4 has a key role in fundamental aspects of cancer, including proliferation, migration, invasion, and angiogenesis (35, 69–76, 234–237), leading to a number of programs to develop therapeutic anti-CXCR4 antibodies. The most advanced candidate is MDX-1338, an anti-CXCR4 mAb also known as BMS-936564 (owned by Bristol-Myers Squibb Co.). It was raised on human Ig transgenic mice immunized with human CXCR4-transfected mouse cells (232). This antibody (IgG4) blocks CXCL12 binding to its receptor with high affinity, and inhibits CXCL12-induced migration and calcium flux. MDX-1338 shows anti-tumoral activity in xenografts of acute myeloid leukemia (AML), non-Hodgkin lymphoma (NHL), and multiple myeloma. *In vitro* assays showed that the antibody triggers tumor cell apoptosis, allowing to propose it as one of the mechanisms of tumor growth inhibition (232).

MDX-1338 is currently undergoing two Phase I studies. The first-in-human study (ClinicalTrials.gov Identifier: NCT01120457) started in 2010 and was planned to be accomplished by the end of 2014 and to enroll up to 82 patients. This anti-CXCR4 mAb is being evaluated as a monotherapy and combined with chemotherapy to treat patients with relapsed/refractory AML, diffuse large B-cell leukemia, chronic lymphocytic leukemia (CLL), or follicular lymphoma. The aim of the trial is to determine the safety, tolerability, maximum tolerated dose, preliminary pharmacodynamics, and efficacy. A second Phase I trial (NCT01359657) started in 2011 to determine safety and tolerability of MDX-1338 as monotherapy or in combination with lenalidomide/dexamethasone or bortezomib/dexamethasone in subjects with relapsed/refractory multiple myeloma. This study is planned to enroll up to 64 patients and be finished in 2015.

Other antibody-derived molecules targeting CXCR4 are being evaluated in clinical trials. This is the case for ALX-0651 (owned by Ablynx, Belgium), a biparatopic anti-CXCR4 nanobody, directed against two different epitopes of CXCR4 (32). Nanobodies are single-domain proteins, derived from the antibody-binding fragment of camelid antibodies. Their immunoglobulins are devoid of light chains and possess only heavy-chains. Nanobodies have the advantages of their relative small size (12–15 kDa) and high solubility, which allows them to cross tissue barriers easily than mammalian immunoglobulins (with a 10-fold higher M.W.). ALX-0651 effectively mobilizes hematopoietic stem cells in a pre-clinical cynomolgus monkey model (32). A Phase I study of safety and effectiveness for this nanobody in healthy volunteers started in 2011 (NCT01374503), but no results have yet been reported.

In addition, many pre-clinical reports have demonstrated the *in vivo* relevance of CXCR4 as a target for cancer therapy. An early report by Müller and co-workers demonstrated a key role for chemokine receptors in metastasis, linking the expression of CXCR4 in breast carcinomas with their ability to generate regional lymph node and lung metastases (35). These data were supported by experiments in which a neutralizing anti-human CXCR4 antibody (clone 44717.111) led to a significant decrease in lung, inguinal, and axillary lymph node metastases. This work highlighted that chemokine interactions with chemokine receptors might turn to be crucial for breast cancer metastasis, showing that antibodies anti-CXCR4 may be useful to interfere with tumor progression and metastasis. Subsequently, similar results were obtained treating xenografts of a human NHL (26) and of a primary human AML (38) with another anti-human CXCR4 antibody (clone 12G5). In both models, a significant reduction on tumor progression was reported. In endometrial cancer xenografts, treatment with 12G5 mAb led to a complete inhibition of spontaneous metastases in liver and lung, and a 28-fold decrease in metastatic index in the peritoneum (30). Interestingly, on an intratibial human osteosarcoma xenograft model, 12G5 mAb reduced metastatic spread to the lung (27).

### CCR2

CCR2 expression in tumor cells facilitates prostate and breast cancer metastases to the bone, where its ligand CCL2 is expressed (155, 238). Prostate cancer patients with bone metastases had higher CCL2 serum levels than patients with localized tumors (155). *In vitro* and *in vivo* experiments using CCR2 or CCL2 knocked down prostate cancer cells demonstrated that these proteins promote prostate cancer growth in bone (238). Similarly, breast cancer metastasis to bone and lung is facilitated by CCL2 interaction with the CCR2^+^ stromal cells of monocytic origin, including macrophages and pre-osteoclasts (238). MLN1202, a humanized, neutralizing anti-CCR2 mAb (224) (developed by Millenium Pharmaceuticals Inc., currently Takeda Pharmaceuticals Co.) went through a Phase II clinical trial for the treatment of bone metastases (NCT01015560). MLN1202 was administered to 44 patients with bone metastasis to address its effect on tumor cell proliferation, monocyte/macrophage trafficking, and osteoclast maturation. Forty-one out of 43 eligible patients completed this study, with 7% having serious adverse events. The concentration in urine of the *n*-telopeptide, a biomarker to measure bone turnover rates, decreased in 14% of the patients after 43 days of MLN 1202 treatment, suggesting a positive effect of the antibody in these patients.

### CCR4

CCR4 is a chemokine receptor predominantly expressed on type 2 T helper cells (Th2), Foxp3^+^ regulatory T-cells (Treg), a subset of CD4^+^ Th17 cells, and skin-homing T-cells positive for cutaneous lymphocyte antigen (CLA) (239–243). This receptor binds two ligands, CCL17 (formerly TARC) and CCL22 (formerly MDC), and has been implicated in the pathology of inflammatory diseases and cancer, being over-expressed on many malignant ATL, CTCL, and PTCL cells (161–163).

The antibody KW-0761 (mogamulizumab), a derivative of the mouse KM2160 mAb, is a humanized defucosylated IgG1 mAb targeting CCR4 (developed by Kyowa Hakko Kirin Co.) (233). KM2160, its chimera KM2760 and the humanized version KW-0761 recognize the N-terminal region of human CCR4 (163, 233, 244). They neither block the interaction between CCR4 and their ligands nor inhibit CCR4 signaling (233). Defucosylation increases Fc-binding to the Fcγ receptors expressed on cytotoxic cells, activating them for tumor cell killing (245). In fact, KM2760 showed potent anti-tumor activity in mouse xenografts of CCR4^+^ cell lines derived from patients diagnosed of ATL, Hodgkin lymphoma, or CTCL (242, 246, 247). In addition, this mAb showed enhanced ADCC against primary CCR4^+^ ATL cells both *in vitro* and *in vivo* in an autologous setting (163, 233, 246). A phase I clinical trial of KW−0761 for patients with relapsed CCR4^+^ PTCL or ATL was the first one to examine the safety and efficacy of a new generation defucosylated therapeutic antibodies for cancer treatment (NCT00355472) (248).

KW-0761 was approved for therapeutic use in Japan for relapsed or refractory CCR4^+^ ATL in 2012, and for relapsed or refractory CCR4^+^ PTCL or CTCL in 2014, representing the first approved antibody drug against GPCR receptors being used for cancer therapy. In other countries, there are several clinical trials currently under way, such as a Phase II (NCT01611142) for patients with relapsed or refractory PTCL, and a Phase III for the comparison of progression-free survival, after treatment with either KW-0761 or vorinostat (a chemical inhibitor of histone deacetylases), on patients with previously treated CTCL (NCT01728805). Despite the positive effects of mogamulizumab in resistant/refractory ATL, PTCL, or CTCL, the application for its use in untreated CCR4^+^ ATL was withdrawn by the company, on February 2014.

A therapeutic potential for mogamulizumab has been suggested against Epstein–Barr virus (EBV)-associated T- and NK-cell lymphoproliferative diseases, which can be refractory to conventional chemotherapies (249). In particular, since this mAb induced ADCC activity against CCR4^+^ EBV^+^-T and -NK-cell lines, and inhibited the growth of EBV^+^ NK-cell lymphomas in xenografts (249).

It is known that Treg cells can facilitate tumor cell evasion from immune surveillance (250). Since CCR4 is expressed on Treg cells, it was conceivable that treatments targeting CCR4 might affect Treg cells. On CTCL patients, a single dose of mogamulizumab has been shown to reduce the fraction of CCR4^+^ malignant T-cells, with a concomitant reduction of CCR4^+^ Treg cells. Interestingly, the reduction of Treg cells may, in turn, improve the immune profiles of these patients (251). KM2760 has also been used, in co-treatment with NK cells, for the *in vitro* elimination of Treg cells (252). A patient treated with mogamulizumab suffered serious adverse reactions and the Stevens–Johnson syndrome (a milder form of toxic epidermal necrolysis), probably due to a significant reduction of its Treg cells (253). A positive interpretation of these results would suggest, however, that anti-CCR4 mAbs could be used on CCR4^−^ tumors to deplete Treg cells from circulation and infiltrating the tumor mass. Indeed, a phase I clinical trial of mogamulizumab for CCR4^−^ solid cancers (UMIN000010050), specifically aiming to deplete Treg cells is currently under way (253). Other adverse effects of mogamulizumab include cutaneous reactions that improve over time (254), the reactivation of hepatitis B virus (255), or diffuse panbronchiolitis (256).

There are other anti-CCR4 mAbs being screened on discovery or pre-clinical phases. Among them, mAb1567 is a humanized neutralizing anti-CCR4 antibody that exhibits potent anti-CCR4^+^ CTCL tumor activity in xenografts, where it displays *in vitro* CDC and neutrophil-mediated ADCC. mAb1567 also exerts *in vitro* human NK cell-mediated ADCC (29). In addition, SCID-beige mice expressing an adenovirus construct derived from mAb1567 allowed an effective *in vivo* treatment of CTCL (257). Furthermore, mAb2–3, an affinity-optimized variant of the humanized mAb1567, has been selected for further pre-clinical development (29). Human anti-CCR4 antibodies, generated by phage display (17G and 9E), also show *in vitro* efficient killing of CCR4^+^ tumor cells via ADCC and phagocytosis, and improved survival in xenografts (31).

## Chemokine Receptors with Antibodies in Discovery or Pre-Clinical Assays for Cancer Treatment

### CXCR2

CXCR2 and its ligands, the chemokines CXCL1, CXCL2, CXCL3, CXCL5, CXCL6, CXCL7, and CXCL8, are expressed by a wide variety of human cancer types (41, 258). CXCR2 is a potent protumorigenic receptor that mediates the recruitment of immunosuppressive leukocytes into tissues, in particular of neutrophils (259). Immunopathological analyses demonstrated the expression of high levels of CXCR2 and its ligand CXCL5 in pancreatic tumors (33). Thus, it was conceivable that treatment with anti-CXCR2 antibodies would inhibit leukocyte infiltration and their pro-tumoral activities. Indeed, treatment with neutralizing anti-mouse CXCR2 antibodies of human pancreatic tumors growing as xenografts displayed reduced tumor volumes, decreased proliferation indexes, and microvessel densities (33, 260). The pancreatic tumor cell line used in that model was devoid of CXCR2 expression (261). CXCR2 can also be expressed on endothelial cells (262), where it can mediate angiogenesis (263). These data, together with a series of *in vitro* assays demonstrating an anti-angiogenic role for antagonist anti-CXCR2 mAbs in endothelial cells (260), suggested that inhibition of tumor growth in the xenograft model was due to angiogenesis inhibition mediated by these antibodies.

### CXCR5

A different approach was used to target CXCR5. In this case, a bispecific Ab, containing paratopes recognizing CXCR5 on the one side and the CD3-T-cell co-receptor on the other, was used (36). With this tool, the authors were able to bring together the tumor cell, recognized with the anti-CXCR5 paratope, with a T lymphocyte, recognized with the anti-CD3 paratope, maintaining the ability of the antibody to bind cells of the innate immune response through the Fc region, potentiating in this way the anti-tumoral response (36). This bispecific antibody was highly efficient lysing tumor cells at low concentrations not only *in vitro* but also *in vivo* on xenograft models of B-cell lymphoma (36).

### CCR7

In subcutaneously injected human mantle cell lymphoma (MCL) cells on mice, anti-CCR7 treatment caused a significant delay on tumor growth and metastasis generation. It also hindered lymphoma cell dissemination in intravenous injections (37). The data obtained were compatible with both decreased infiltration of MCL cells into different tissues and the induction of anti-MCL cell cytotoxicity in mice. Anti-CCR7 therapy might be indicated for patients carrying CCR7^+^ B-cell NHL or CLL (37).

### CCR9

We have reported the generation and characterization of 91R, a mouse anti-human CCR9 mAb able to reduce 85% human T lymphoblastic cell tumors on mice. Tumor size reduction was concomitant with an increased apoptotic tumor cell fraction and tumor necrotic areas, as well as decreased fraction of proliferating cells and tumor vascularization. It is likely that CDC or ADCC represent the *in vivo* mechanisms of action of this mAb (28). These results suggest that CCR9-expressing tumors, such as acute and chronic T-cell lineage leukemia (180), prostate cancer (181), breast cancer (182), and melanomas (183), can potentially be targeted with this mAb.

### ACKR3 (formerly CXCR7)

Anti-ACKR3 nanobodies inhibited tumor growth of ACKR3^+^ head and neck cancer cells, reducing expression of the endothelial cell marker CD31 in the tumors growing as xenografts. These data were corroborated by *in vitro* analyses demonstrating that anti-ACKR3 nanobodies rather than affecting cell cycle progression, reduced the secretion of the angiogenic chemokine CXCL1 by the tumor cells, suggesting that anti-ACKR3 nanobodies could inhibit tumor vascularization. This work proposes anti-ACKR3 therapies as potential novel treatments against ACKR3^+^ head and neck cancer (34).

The antibodies described so far are representative of the use of anti-chemokine receptor antibodies for cancer treatment, but they represent only the tip of the iceberg, since many companies describe in their web pages, or have already presented in specialized meetings their efforts for developing new antibodies. Some of these antibodies are AT008 (anti-CCR4) and AT009 (anti-CXCR4) from Affitech; anti-CXCR5, anti-CCR2, and anti-CXCR3 from Sorrento Therapeutics Inc.; anti-CXCR4 (515H7) from Pierre Fabre (264); and anti-CXCR4 (CX-02 and CX-05) from NorthWest Biotherapeutics Inc. (265).

## Conclusion and Perspectives

Chemokines and their receptors, in addition to their role on physiological responses, directing the cells toward specific sites, allowing lymphocyte maturation, survival, proliferation, and migration, they play a key role in cancer initiation, angiogenesis, tumor growth, progression, and metastasis. The over-expression by many tumors of chemokine receptors turns them and their ligands into clear targets for cancer therapy, on the initial assumption that inhibition of chemokine signaling might block tumor progression and/or metastasis.

The development of small molecules able to inhibit chemokine receptor signaling has shown limited success, with the exception of plerixafor (AMD3100), which by blocking binding of CXCL12 to CXCR4 mobilizes CXCR4^+^ cells, including CXCR4^+^ tumor cells, from the bone marrow to circulation (266, 267). Plerixafor enhances sensitivity of tumor cells to cytotoxic agents by disrupting interaction with the tumor microenvironment (268–271). Since therapeutic mAbs have provided clinical benefits to cancer patients during the last decade, therapeutic antibodies against chemokine receptors seemed a good alternative. The recent technological advances allowed the generation of highly specific, high affinity antibodies against these receptors that are currently entering the clinics.

This “theoretical” view suggesting that anti-chemokine receptor mAbs might represent an efficient way to treat cancer was fully supported by the recent approval in Japan of mogamulizumab for the treatment of ATL. It is so far the only anti-GPCR therapeutic antibody in the market and represents the proof-of-principle for the therapeutic use of mAbs targeting GPCRs, as on chemokine receptors, leading to clinical benefits on cancer patients.

Chemokine receptors are expressed, in addition to endothelial cells, on the tumor and on cells from the immune system, responsible for defending from the tumor. Therefore, any drug or antibody targeting a given chemokine receptor will act on both the tumor and immune system cells expressing it. In the case of mogamulizumab, raised against CCR4, it should be noted that there is only a fraction of T lymphocytes (within the Th2, Treg, and Th17 phenotypes) that are CCR4^+^, representing the few non-tumoral targets for this antibody. Since the phenotypes affected include Treg and Th17 cells, their elimination would become advantageous for treating the tumor, as the immunosuppressive responses would be reduced. Treatment with antibodies against other chemokine receptors such as CXCR4 or CCR7 might have a broad effect on the host immune response, since there is a large fraction of leukocytes expressing them. This effect is not necessarily beneficial in terms of reducing tumor size.

These data raise the question whether other anti-chemokine receptors can be safely used as targets for tumor treatment. In particular, it should be determined, for each chemokine receptor, whether there are therapeutic doses of an antibody able to effectively kill tumor cells or cells favoring tumor growth, while not affecting the normal cells from the immune system expressing that particular receptor. In other words, the aim is to find the therapeutic window where tumor cells are safely destroyed whereas the immune cells are not. This can be exemplified with data on CCR2^+^ tumor infiltrating cells, where if the infiltrating cells are macrophages supporting the metastatic dissemination of malignant cells, the anti-CCR2 treatment may be effective. Conversely, if the infiltrating cells are CD8^+^ and γδ effector T-cells that enhance immuno-surveillance by triggering Th1 responses, the treatment might turn deleterious (272, 273).

Thus, the complex network between chemokine receptors and their ligands, together with the simultaneous expression of a given receptor on cells of the immune system and on the tumor (including tumor cells, stroma cells, and/or tumor infiltrating cells) and the dichotomy of their responses clearly represent the lights and shadows of the potential of anti-chemokine receptor therapeutic antibodies for the treatment of tumors. The optimal situation would be to have a panel of therapeutic antibodies against different chemokine receptors. These antibodies could be used, depending on the tumor phenotype, in particular combinations (personalized medicine) at relatively low doses for each one of them, minimizing the probability of affecting the normal cells.

## Conflict of Interest Statement

The authors declare that the research was conducted in the absence of any commercial or financial relationships that could be construed as a potential conflict of interest.
